# Intermethod Association in Orthodontic White Spot Lesion Assessment: A Multimodal-Assessment

**DOI:** 10.1016/j.identj.2026.109740

**Published:** 2026-07-17

**Authors:** Ezgi Cansu Firinciogullari, Genta Agani Sabah, Aslıhan Mediha Erdinc, Burcu Uner

**Affiliations:** a Department of Applied Craniofacial Sciences, Medical University of South Carolina, Charleston, South Carolina, USA; b Department of Orthodontics, Faculty of Dentistry, Izmir Tinaztepe University, Izmir, Türkiye; c Department of Orthodontics, Ege University School of Dentistry, Izmir, Türkiye; d University of Nebraska Medical Center, Omaha, Nebraska, USA

**Keywords:** White spot lesions, Remineralization, Quantitative light-induced fluorescence, DIAGNOdent, SEM–EDS, Orthodontics

## Abstract

**Aims:**

To evaluate stage-specific intermethod association among DIAGNOdent, quantitative light-induced fluorescence (QLF), scanning electron microscopy with energy-dispersive X-ray spectroscopy (SEM–EDS), and colourimetry in white spot lesion (WSL) assessment, and to compare alternative explanatory model structures for QLF-derived lesion volume (Δ*Q*).

**Methods:**

This secondary in vitro matched-block analysis included 20 bovine incisors sectioned into four enamel surfaces (80 surfaces). Within each tooth, surfaces were allocated to one of four groups. Measurements were obtained at baseline (T0), postdemineralization (T1), day 7 (T2), and day 30 post-treatment (T3). DIAGNOdent, QLF metrics (Δ*F*, lesion area [LA], and Δ*Q*), colourimetry, and SEM–EDS values were recorded. Statistical analyses included Spearman correlation and mixed-effects models.

**Results:**

At T1, intermethod association was limited, with weak associations among QLF, SEM–EDS, and DIAGNOdent. At T3, stronger associations were observed, particularly between intensity-based QLF metrics and mineral-related parameters. Ca/P showed positive associations with Δ*F* and Δ*Q*, while LA was inversely associated with Ca/P. DIAGNOdent scores were inversely associated with Ca/P and Δ*F* and positively associated with LA. Mixed-effects analyses identified a limited number of significant predictors of Δ*Q*, and the combined model demonstrated better relative in-sample fit than the clinical and compositional models at both T1 and T3.

**Conclusions:**

WSL assessment methods provided complementary rather than interchangeable information. Cross-method associations were limited after lesion induction, whereas stronger associations were observed after treatment, particularly between intensity-based QLF parameters and mineral-related measures. No single metric fully characterized WSL change, and the combined model showed modest explanatory performance.

**Clinical relevance:**

No single chairside measure appears sufficient for all aspects of WSL assessment. Within the limits of this in vitro study, intensity-based QLF measures were more closely aligned with mineral-related changes after treatment, whereas LA and colour-based indices provided complementary information on lesion extent and visible appearance.

## Introduction

White spot lesions (WSLs) are the earliest clinically detectable stage of enamel caries and remain a frequent and aesthetically relevant complication, particularly in patients with increased plaque retention, such as those undergoing orthodontic treatment.^1^^,^^2^ Current management emphasizes nonoperative strategies that restore the demineralization–remineralization balance.^3^ In the remineralization process, mineral deposition may be more pronounced at the surface and may not fully reflect recovery of deeper lesions. Consequently, WSL research and monitoring commonly employ multiple measurement modalities that capture complementary physical aspects of lesion status.^4^

DIAGNOdent provides a rapid, clinically convenient laser-fluorescence signal that correlates with lesion activity. Quantitative light-induced fluorescence (QLF) provides lesion-centred metrics, including fluorescence loss (Δ*F*), lesion area (LA), and lesion volume (Δ*Q*), which is defined as the fluorescence loss integrated over the LA. Scanning electron microscopy with energy-dispersive X-ray spectroscopy (SEM–EDS) provides local elemental composition endpoints (Ca and P weight percentages, and the Ca/P ratio), while colorimetric measurements quantify optical appearance, including the Whiteness Index for Dentistry (WID).^4^, ^5^, ^6^, ^7^ Although these modalities are widely used, it is not always clear whether clinically accessible measures, such as laser fluorescence and colour, can serve as informative proxies for more mechanistic outputs, such as QLF lesion volume or EDS-derived mineral recovery.

The physical bases of these modalities differ in ways that are likely to produce stage-specific cross-method association patterns. In the present study, cross-method alignment is defined operationally as the strength and direction of associations among measures obtained from different modalities within the same lesion stage, rather than as agreement in the strict sense of interchangeability or predictive capacity. QLF primarily reflects fluorescence loss associated with subsurface porosity and altered light propagation, whereas SEM–EDS provides localized near-surface elemental information. DIAGNOdent and colorimetric measures may respond to surface-related optical changes that do not map directly onto subsurface mineral status.^4^ Accordingly, intermethod association may be limited during early lesion formation, when subsurface optical changes may precede detectable shifts in near-surface composition, and may increase during recovery, as mineral infilling brings optical and compositional signals into closer alignment. Whether this stage-specific convergence pattern is empirically detectable across these four modalities within the same matched experimental framework forms the basis of the present study.

The primary objective of this study was, therefore, to evaluate intermethod associations among four assessment systems (DIAGNOdent, QLF, SEM–EDS, and colourimetry) across lesion formation and recovery stages in a matched-block in vitro design. Specific aims included: (1) characterizing intermethod associations at postdemineralization and post-treatment stages; (2) estimating key intermethod slopes using mixed-effects models that account for within-tooth clustering; (3) evaluating the in-sample explanatory performance of models predicting QLF-derived lesion volume from clinically accessible measures; and (4) comparing competing model structures using information criteria. The study hypothesis was that intermethod association would show different patterns across lesion stages, with weaker cross-method associations after lesion induction and stronger associations during the post-treatment stage.

## Material and methods

*Study design and experimental unit:* This study is a secondary analysis of a previously reported in vitro dataset. The primary study was designed to evaluate the remineralization capacity of different agents within individual assessment domains.^4^ In contrast, the present secondary analysis addresses a distinct research question by examining stage-specific cross-method associations across DIAGNOdent, QLF, SEM–EDS, and colourimetry within the same matched-block dataset. The novel components of this analysis include time-point-specific intermethod correlation matrices, mixed-effects modelling of selected cross-method relationships, and a comparative evaluation of alternative multivariable model structures involving QLF-derived lesion burden as an operational lesion-related outcome. The sample size was determined for the primary study and was not recalculated for the present analysis. Full methodological details regarding specimen preparation, demineralization protocol, and remineralization procedures are described in the primary report, and key procedural elements are summarized below for completeness. The dataset was acquired using four measurement modalities (DIAGNOdent, QLF, SEM–EDS, and colourimetry) under a matched-block design, with the tooth (ToothID) as the matching unit. Four enamel surfaces from each tooth were assigned to four treatment groups. Analyses were planned to account for within-tooth clustering and repeated measurements over time.

*Specimen preparation and allocation (matched blocks):* A total of 20 bovine incisors were used. Each tooth was sectioned in mesiodistal and occlusogingival directions to obtain four enamel surfaces (80 surfaces total). Within each tooth, one surface was assigned to each group (*n* = 20): Group 1: control (no treatment); Group 2: fluoride varnish (Voco Profluorid 5% NaF Varnish, VOCO Dental, Cuxhaven); Group 3: CPP-ACFP (MI Paste Plus with Recaldent, GC Corporation); and Group 4: P11-4F, fluoride-containing self-assembling peptide (CURODONT Repair Fluoride Plus, Credentis AG). A standardized central measurement window was defined on each surface, and all device measurements were obtained from the same window across time points.

*Demineralization and remineralization:* WSLs were created by immersing the specimens in 40 mL of a demineralizing solution (pH 4.3; 2.0 mmol/L calcium, 2.0 mmol/L phosphate, 75 mmol/L acetate) at 37°C for 6 hours, followed by immersion in 20 mL of a remineralizing solution (pH 7.0; 1.5 mmol/L calcium, 0.9 mmol/L phosphate, 150 mmol/L potassium chloride, 20 mmol/L cacodylate buffer) at 37°C for 18 hours. Measurements were obtained at T0 (sound enamel baseline), T1 (postdemineralization WSL), T2 (day 7 post-treatment), and T3 (day 30 post-treatment). Remineralization agents were applied after T1 according to the manufacturer’s instructions, and specimens were stored under the same conditions throughout the follow-up period, aligning with the protocol used in recent research.

*Outcome measures:* To structure the analysis, QLF-derived intensity-based metrics (Δ*F* and Δ*Q*) were treated as indicators of fluorescence loss severity, and LA was treated as a descriptor of lesion footprint. This distinction is operational and reflects differences in what each metric quantifies within the QLF domain; it is not proposed as a validated biological classification of remineralization compartments. These metrics were evaluated in parallel across treatment stages using the following four modalities:

• *Laser fluorescence:* DIAGNOdent 2095 readings were recorded at T0, T1, T2, and T3. Two change scores were computed: ΔDIAGNOdent (T0 → T1) and ΔDIAGNOdent (T1 → T3).
• *Colour measurements (CIELAB, WID):* Colour was measured at four time points using a digital spectrophotometer (Vita EasyShade, Vita Zahnfabrik) based on the CIE Lab* system. For each surface, measurements were repeated three times and averaged to obtain L*, a*, and b*. The WID was computed as WID = 0.55·L* – 2.32·a* – 1.100·b*, and whiteness change between time points was computed as ΔWID.
• *SEM–EDS elemental analysis:* Using a scanning electron microscope with an EDS X-ray detector (KaVo Dental), the Ca and P weight percentages and Ca/P ratio for each surface at four time points were recorded.
• *QLF:* QLF images were acquired using a standardized in vitro imaging setup (QLF-D Biluminator) and analysed with the device software to obtain intensity-based metrics (Δ*F* and Δ*Q*) and LA. QLF metrics were available and analysed at T0, T1, and T3. QLF fluorescence-loss outputs are exported as negative values; throughout, native-sign QLF outputs are reported, where values closer to zero indicate less fluorescence loss. In this study, QLF-derived parameters were conceptually grouped into two categories: intensity-based fluorescence metrics (eg, Δ*F* and Δ*Q*), which reflect fluorescence loss and the magnitude of lesion-related optical change, and lesion footprint metrics (eg, LA), which represent the spatial extent of the lesion.

The analytic framework of this secondary analysis was structured into three components. The primary, prespecified objective was to evaluate cross-method associations across lesion stages. Within this framework, Δ*Q* was selected as the modelled QLF outcome, serving as a structured, domain-specific summary measure of lesion burden within the QLF domain, rather than as a reference standard. Model-based comparisons (clinical, compositional, and combined models) were prespecified as secondary analytical components to represent distinct domains of information and to examine their relative explanatory performance. Additional analyses, including sensitivity and stratified models, were considered exploratory and are interpreted accordingly. This analytical structure was defined to organize the interpretation of the existing dataset rather than to select models post hoc on the basis of the most interpretable findings.

## Statistical analysis

Prior to analysis, the dataset was converted to long format, with each observation indexed by ToothID (representing the matched block), Group (four levels), Time (T0-T3), and the corresponding outcome variables. Although 80 surfaces were available for analysis, surfaces were nested within 20 teeth (four surfaces per tooth), and the tooth constituted the independent sampling unit. Mixed-effects models with ToothID as a random intercept were used throughout to account for this within-tooth clustering. Accordingly, the effective number of independent units for all analyses was 20. QLF outcomes were analysed only at time points where imaging was performed (T0, T1, and T3). The T2 time point was designated in the primary study protocol as an interim clinical observation. QLF imaging was not performed at this visit, and QLF outcomes were therefore not modelled at T2.

Analyses were organized to address three prespecified objectives: (1) characterizing intermethod associations at postdemineralization and post-treatment stages; (2) estimating key intermethod slopes using mixed-effects models; and (3) evaluating the comparative explanatory performance of single-domain vs multimodal model structures for QLF-derived lesion volume (Δ*Q*).

Intermethod associations were examined at T0, T1, and T3, as well as for change scores (ΔT1 → T3). Pairwise associations were summarized using Spearman’s rank correlation (*ρ*), interpreted using prespecified thresholds: |*ρ*| < 0.30 as weak, 0.30 to 0.59 as moderate, and ≥ 0.60 as strong. Multiple testing was controlled using the Benjamini–Hochberg false discovery rate (FDR) procedure, applied within each time-point–specific correlation matrix (T0, T1, T3) and within the change-score matrix (ΔT1 → T3). Statistical significance was evaluated at *α* = 0.05. Spearman correlations were used as exploratory inferential analyses to assess cross-method associations within each stage. Although FDR-adjusted q-values are reported to control for multiple comparisons, these analyses are interpreted within an exploratory rather than confirmatory framework.

Mixed-effects models with ToothID as a random intercept were used to estimate slope coefficients (*β*) and 95% confidence intervals for key intermethod relationships, with Δ*Q* as the primary outcome. Random-slope structures were explored but were not retained due to convergence instability in some specifications, consistent with the limited number of independent clusters (*n* = 20 teeth). For model-structure comparison, a clinical model (DIAGNOdent, L, WID), a compositional model (Ca/P ratio), and a combined model (all predictors) were fitted and compared using AIC/BIC. The clinical model includes measures obtainable without destructive or laboratory-based analysis; the compositional model includes the mineral-balance indicator derived from SEM–EDS; and the combined model integrates predictors from both domains. In-sample explanatory performance was evaluated by reporting *R*², RMSE, and MAE for observed vs model-predicted Δ*Q*. These metrics reflect model fit within the present dataset and should not be interpreted as estimates of external predictive validity or individual-level prediction accuracy. Model robustness was assessed using sensitivity analyses, including group-adjusted and treatment-stratified models. To evaluate stage-dependence of selected intermethod associations, Fisher’s *r*-to-*z* transformation tests were used to compare Spearman correlation coefficients between T1 and T3 for four prespecified pairs. Statistical significance was evaluated at *α* = 0.05. All analyses were performed in R (version 4.5.2) within RStudio (version 2026.01.1-403). Reproducible code and computational details are provided in the Supplementary Materials.

## Results

**Fig. 1:**
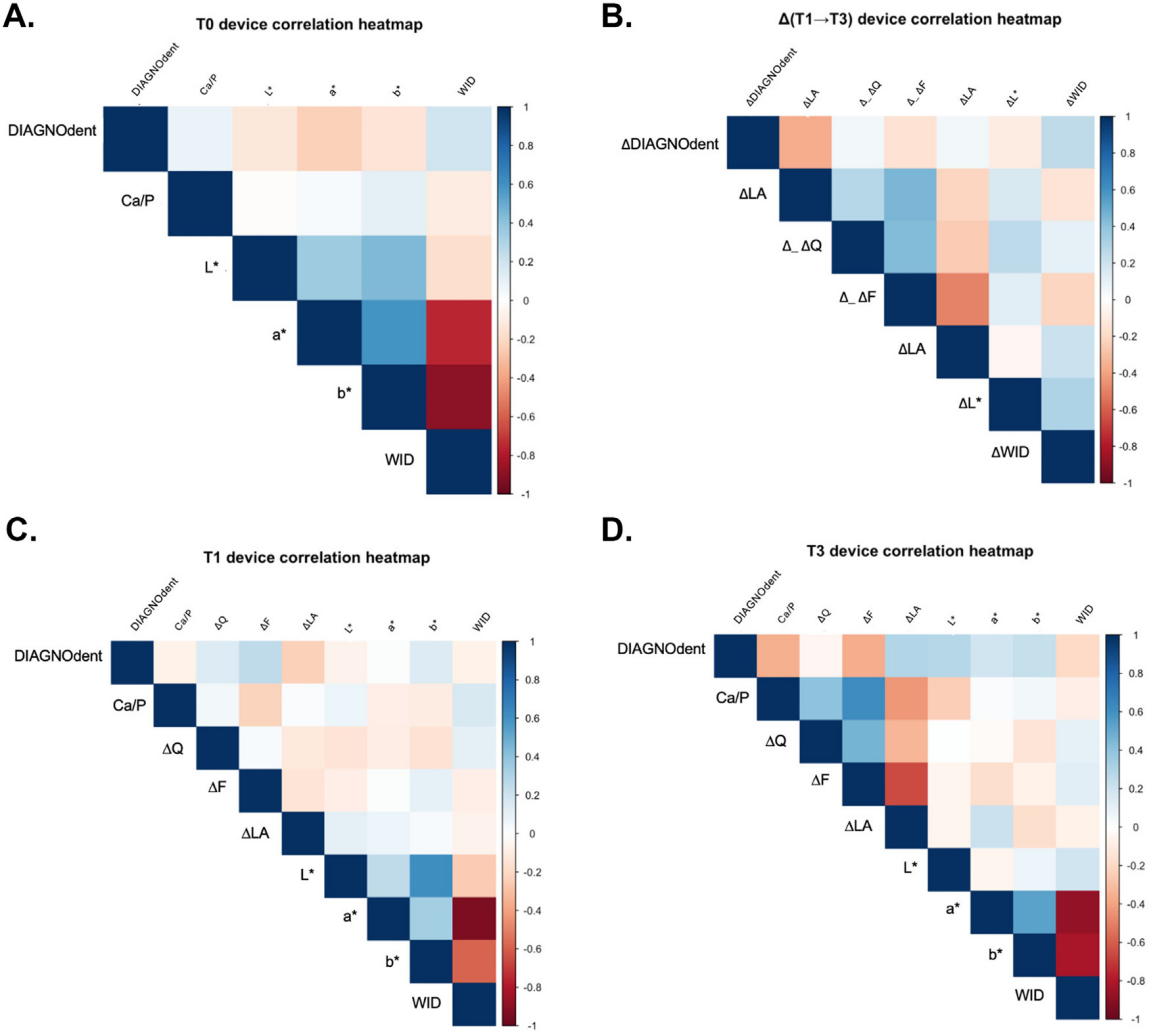
Heatmap of correlations. Colour intensity indicates the strength of the correlation, ranging from −1 (negative association) to +1 (positive association). Positive values indicate that measures tend to vary in the same direction, whereas negative values indicate opposite directional change. Stronger associations indicate greater cross-method alignment within that stage, whereas weaker associations suggest the modalities may capture different lesion-related features. (A) T0 device correlation heatmap, (B) Δ(T1→T3) device correlation heatmap, (C) T1 device correlation heatmap, (D) T3 device correlation heatmap.

At baseline (T0; Figure 1A), correlations were largely confined to the colour domain. Strong associations were observed between a* and b* (*ρ* = 0.596, *q* < 0.001), and WID showed strong negative correlations with both b* (*ρ* = −0.897, *q* < 0.001) and a* (*ρ* = −0.756, *q* < 0.001). L* was moderately positively associated with both a* (*ρ* = 0.357, *q* < 0.001) and b* (*ρ* = 0.442, *q* < 0.001), whereas DIAGNOdent and Ca/P showed no meaningful association (*ρ* = 0.083, *q* = 0.756).

After lesion induction (T1; Figure 1B), QLF lesion metrics became measurable. QLF internal associations at T1 were weak: Δ*Q* and Δ*F* showed near-zero correlation (*ρ* = 0.035), and both showed weak negative associations with LA (Δ*Q*–LA: *ρ* = −0.119; Δ*F*–LA: *ρ* = −0.150). Intermethod associations between QLF and SEM–EDS were similarly small (Ca/P–Δ*Q: ρ* = 0.060; Ca/P–Δ*F: ρ* = −0.230). DIAGNOdent showed weak associations with QLF metrics at T1, including DIAGNOdent–Δ*Q* (*ρ* = 0.142) and DIAGNOdent–Δ*F* (*ρ* = 0.251).

At post-treatment (T3; Figure 1C), QLF internal associations strengthened. Δ*F* and Δ*Q* showed a moderate positive association (*ρ* = 0.463, *q* < 0.001), and both showed negative associations with LA: Δ*F*–LA (*ρ* = −0.655, *q* < 0.001) and Δ*Q*–LA (*ρ* = −0.331, *q* = 0.012). Intensity-based fluorescence metrics aligned with Ca/P at T3 (Ca/P–Δ*F: ρ* = 0.629, *q* < 0.001; Ca/P–Δ*Q: ρ* = 0.405, *q* = 0.0018). The LA showed a moderate inverse association with Ca/P (*ρ* = −0.433, *q* < 0.001). DIAGNOdent showed inverse associations with Ca/P (*ρ* = −0.352, *q* = 0.0073) and with Δ*F* (*ρ* = −0.361, *q* = 0.0064), and a positive association with LA (*ρ* = 0.296, *q* = 0.027). The directionality of associations between Ca/P and clinically accessible measures became more pronounced at T3 than at T0 or T1.

Formal Fisher’s *r*-to-*z* comparisons between T1 and T3 indicated significant stage-dependent differences. The Ca/P–Δ*F* association changed from *ρ* = −0.230 at T1 to *ρ* = 0.629 at T3 (*z* = −6.044, *q* < 0.001). The Ca/P–Δ*Q* association increased from *ρ* = 0.060 at T1 to *ρ* = 0.405 at T3 (*z* = −2.294, *q* = 0.022). The DIAGNOdent–Δ*F* association reversed direction across stages, from *ρ* = 0.251 at T1 to *ρ* = −0.361 at T3 (*z* = 3.940, *q* < 0.001). Similarly, the LA–Ca/P association shifted from *ρ* = 0.024 at T1 to *ρ* = −0.433 at T3 (*z* = 3.024, *q* = 0.003) (Supplementary Table S1).

Treatment-response analyses used change scores from T1 to T3 (Figure 1D). QLF change variables showed moderate internal concordance: Δ*Q* and Δ*F* were moderately associated (*ρ* = 0.434, *q* < 0.001). Δ*F* showed a moderate inverse association with LA (*ρ* = −0.502, *q* < 0.001), while the Δ*Q*–LA association was weaker (*ρ* = −0.253, *q* = 0.0805). Mineral recovery tracked intensity-based change: Ca/P correlated with Δ*F* (*ρ* = 0.451, *q* < 0.001) and showed a weaker association with Δ*Q* (*ρ* = 0.286, *q* = 0.052). DIAGNOdent varied inversely with Ca/P (*ρ* = −0.362, *q* = 0.0094). Colorimetric change variables showed limited coupling with mineral recovery. Meanwhile, within the colorimetric domain, L* and WID change scores were positively associated (*ρ* = 0.308, *q* = 0.039).

**Fig. 2:**
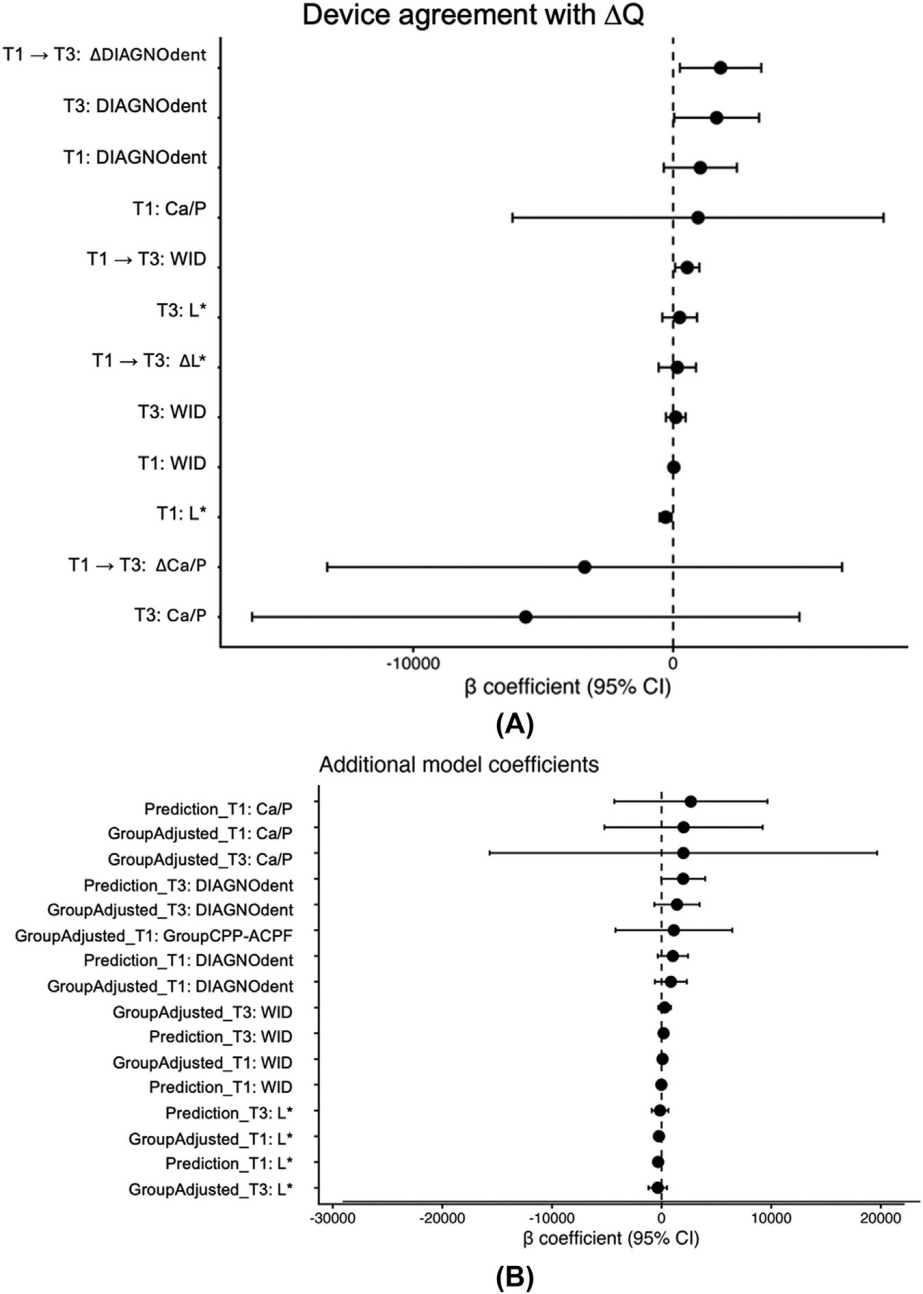
Forest plot showing mixed-effects regression estimates for the association between diagnostic measurements and Δ*Q*. (A) Device agreement with Δ*Q*, (B) Additional Model Coefficients.

In the mixed-effects model for the Δ*Q* outcome, only L* at T1, DIAGNOdent at T3, ΔDIAGNOdent, and ΔWID were significant predictors (Figure 2A; Table 1). In extended and sensitivity analyses, most coefficients were centred near zero and had wide confidence intervals, indicating limited stability of many individual predictor effects after accounting for within-tooth clustering (Figure 2B).

**Table 1:** Mixed-effects results (Δ*Q* outcome; ToothID random intercept).

Time	Predictor	Beta	95% CI (lower to upper)	*P*
T1	DIAGNOdent	1046.22	−362.57 to 2455.01	.151
T1	Ca/P	955.91	−6183.66 to 8095.48	.794
T1	WID	22.02	−101.08 to 145.12	.727
T1	L*	−291.81	−523.70 to −59.93	**.016**
T3	DIAGNOdent	1670.82	38.63 to 3303.01	**.049**
T3	Ca/P	−5673.02	−16203.73 to 4857.69	.296
T3	WID	99.48	−280.42 to 479.39	.610
T3	L*	250.05	−419.16 to 919.26	.467
ΔT1 → T3	ΔDIAGNOdent	1824.32	257.60 to 3391.04	**.025**
ΔT1 → T3	ΔCa/P	−3406.36	−13313.09 to 6500.37	.503
ΔT1 → T3	ΔWID	540.38	74.10 to 1006.66	**.026**
ΔT1 → T3	ΔL*	160.43	−558.93 to 879.78	.663

Bold values indicate statistical significance (*P* < .05).

**Fig. 3:**
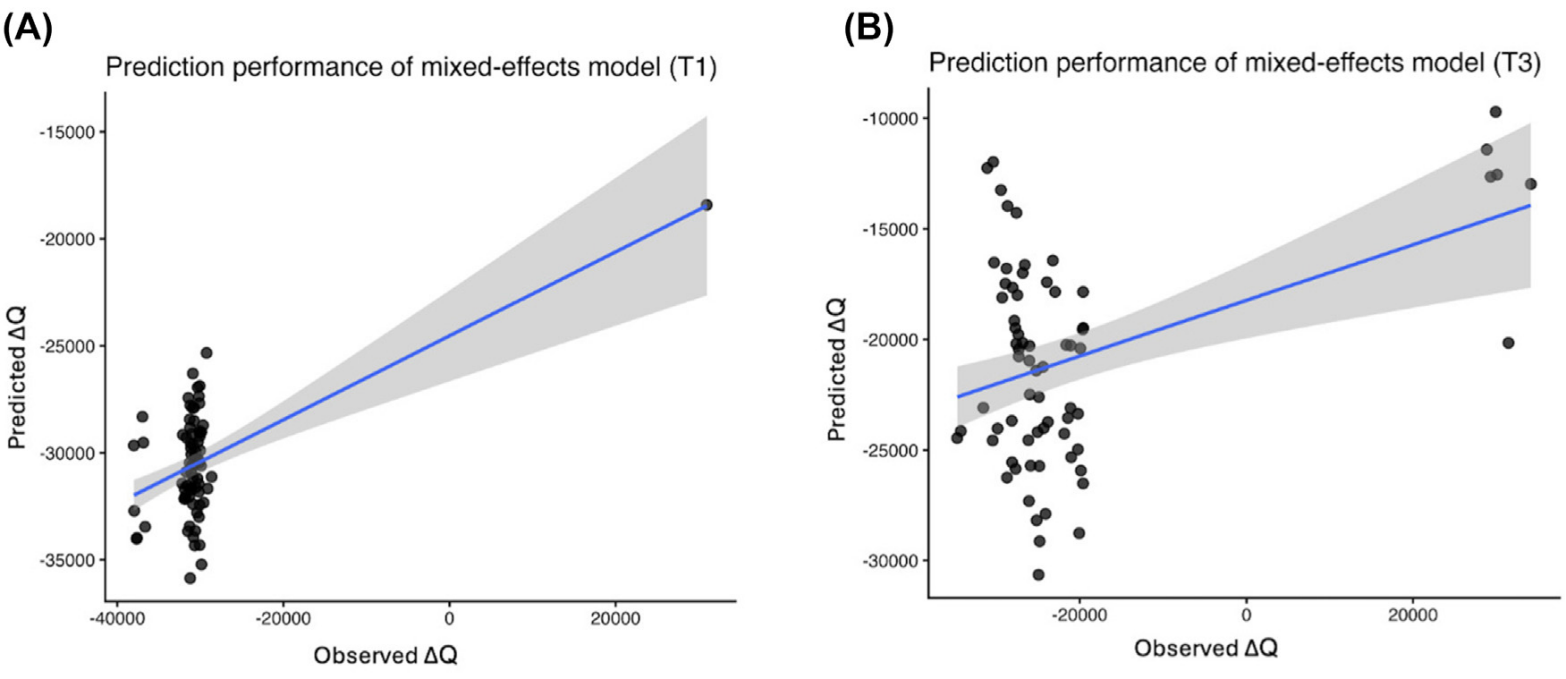
Prediction performance of mixed-effects models. (A) Observed vs predicted Δ*Q* at T1, (B) observed vs predicted Δ*Q* at T3.

**Fig. 4:**
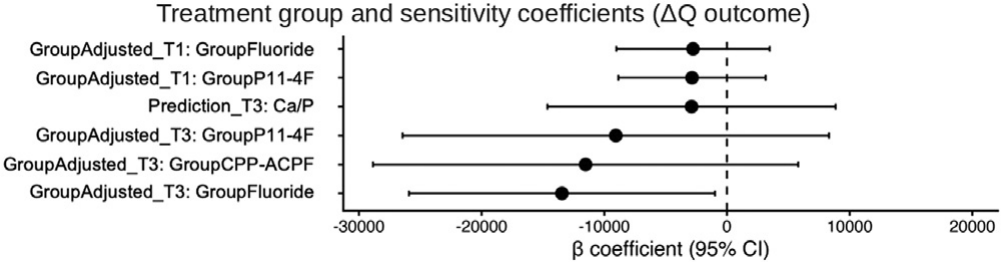
Extended and group-adjusted coefficients for the Δ*Q* outcome.

Model comparison consistently favoured the combined model over the clinical and compositional models at both T1 and T3 (Table 2). Predictive performance for Δ*Q* was modest overall and was better at T1 than at T3, with *R*² = 0.314, RMSE = 6358.19, and MAE = 2774.55 at T1, compared with *R*² = 0.180, RMSE = 14,779.16, and MAE = 9225.49 at T3 (Figure 3A, B). In treatment-stratified analyses, only two predictors reached significance, both within the CPP-ACFP group, whereas all other stratified effects were nonsignificant (Figure 4).

**Table 2:** Model-structure comparison (AIC/BIC).

Stage	Model	AIC	BIC
T1	Clinical	1502.94	1516.52
T1	Compositional	1544.08	1553.30
T1	Combined	1486.18	1501.92
T3	Clinical	1503.11	1516.25
T3	Compositional	1570.47	1579.47
T3	Combined	1485.65	1500.87

## Discussion

This secondary matched-block analysis provides a structured view of how commonly used WSL assessment modalities relate to one another across lesion formation and post-treatment stages. By examining four measurement systems using a within-tooth design, the study suggests that fluorescence-based signals, optical appearance, and near-surface elemental composition provide complementary rather than fully interchangeable information. Rather than merely showing that these modalities assess different properties, the findings suggest that cross-method associations vary across stages. Overall, cross-method alignment was limited after lesion induction, whereas stronger and more coherent associations were observed after treatment. The present analyses were designed to characterize stage-specific patterns of cross-method association, and formal Fisher’s *r*-to-*z* tests were additionally performed for four prespecified intermethod pairs to evaluate whether the observed contrasts between T1 and T3 reflected statistically significant stage-dependent differences. All four pairs showed significant differences after FDR correction, providing formal support for the central hypothesis. These formal comparisons are reported in the Results and summarized in Supplementary Table S1 and are interpreted within the overall exploratory framework of the study.

At baseline, in the parent dataset, Ca and P covaried as expected for hydroxyapatite tissue. In the present secondary analysis, Ca/P was used as the summary compositional indicator because it provides a more stable and interpretable summary of mineral balance within the present cross-method framework. The observation that Ca/P appeared to track calcium more closely than phosphorus can be explained by the variance structure of the dataset. When phosphorus varies over a narrower range, calcium becomes the dominant contributor to between-surface differences in the ratio.^8^, ^9^, ^10^, ^11^, ^12^, ^13^ By contrast, the optical variables demonstrated a more internally coherent structure at baseline, with moderate-to-strong associations among L*, a*, b*, and WID. However, the limited cross-domain coupling between optical variables and compositional indicators, together with the negligible association between DIAGNOdent and Ca/P, suggests that the visible appearance of sound enamel is influenced more by superficial scattering behaviour and intrinsic optical properties than by small differences in near-surface elemental ratios.

After lesion induction, fluorescence-based signals became measurable, yet association across methods remained limited. At T1, the weak internal relationships among QLF metrics, the near-zero association between Δ*Q* and Δ*F*, and the limited associations between fluorescence variables and LA suggest that these parameters were not capturing fully overlapping aspects of early lesion behaviour. Likewise, the small associations between QLF and SEM–EDS measures, together with the weak links between DIAGNOdent and QLF outcomes, support the view that early cross-method convergence was modest. This pattern may partly reflect the restricted variance imposed by a standardized demineralization protocol, but it is also consistent with the fact that the included methods rely on different physical principles and sampling domains. Importantly, this spatial and methodological heterogeneity is not a limitation to be eliminated, but rather a defining feature of the present study, which was designed to examine how fundamentally different measurement domains relate to one another rather than to assess concordance between methods measuring the same construct. QLF primarily reflects fluorescence loss associated with subsurface porosity and altered light scattering,^14^ whereas SEM–EDS provides localized near-surface elemental information.^15^ Accordingly, early lesions may become optically detectable without showing a proportional change in localized near-surface composition.^16^ Clinically, this suggests that no single metric is likely to capture all relevant features of early lesion formation.

Following treatment, the observed pattern was one of greater cross-method association. This increased alignment was most evident between intensity-based fluorescence parameters and compositional recovery. The stronger internal coherence between Δ*F* and Δ*Q* at T3, their inverse relationships with LA, and their closer alignment with Ca/P suggest that intensity-based fluorescence metrics may become more reflective of mineral-associated recovery during the post-treatment phase. By contrast, the only moderate relationship between LA and Ca/P indicates that lesion footprint does not necessarily change in parallel with mineral-associated recovery. Because post-treatment observations were obtained after different interventions, the pooled T3 associations should not be interpreted as reflecting stage-related variation alone; treatment-specific heterogeneity may also have contributed to the observed post-treatment patterns. The inverse associations of DIAGNOdent with Ca/P and Δ*F*, together with its positive association with LA, further suggest that this measure may provide directionally meaningful information regarding post-treatment lesion status. To clarify the directional logic of these associations, it is necessary to consider the sign conventions of each modality explicitly. DIAGNOdent scores are expressed as positive integers in which higher values reflect greater lesion-associated laser fluorescence, conventionally interpreted as indicative of more active or more extensive demineralization; remineralization is therefore expected to produce falling DIAGNOdent scores. QLF-derived fluorescence metrics, by contrast, are exported as negative values in which the magnitude of negativity reflects the degree of fluorescence loss relative to sound enamel; accordingly, values closer to zero represent reduced fluorescence loss and are consistent with less severe or recovering lesions, such that remineralization is expected to produce rising, that is, less negative, Δ*F* values. Within this framework, the observed inverse association between DIAGNOdent and Δ*F* reflects directionally concordant rather than conflicting behaviour: as lesion severity decreases, DIAGNOdent scores fall while QLF Δ*F* values rise towards zero, both consistent with the expected biological direction of remineralization. This sign-convention difference should be borne in mind when interpreting any cross-modality association involving QLF outputs throughout the present analysis. Taken together, these findings support the interpretation that subsurface optical recovery and near-surface compositional change may emerge concurrently during remineralization, but not necessarily at the same rate as measurable reduction in lesion footprint.^17^, ^18^, ^19^, ^20^, ^21^

One of the main conceptual findings of this study is that reductions in optical lesion intensity and reductions in lesion extent do not appear to represent identical aspects of recovery. In the treatment-response analyses, Δ*F* showed stronger relationships than Δ*Q* with both LA and Ca/P, suggesting that mineral-associated recovery may be more closely aligned with reduced fluorescence-loss intensity than with geometric lesion shrinkage alone. Conversely, the more limited associations between colorimetric variables and mineral-related measures indicate that visible esthetic improvement should not be interpreted as a direct surrogate for structural recovery. In other words, a lesion may become less visible, fluorescence loss may decrease, or mineral-related parameters may improve, but these changes may not occur to the same extent or at the same rate. Interpreting lower fluorescence loss, smaller LA, and improved visual appearance as a single uniform construct of ‘recovery’ may therefore oversimplify the multidimensional nature of WSL repair.^18^^,^^19^

Δ*Q* was selected as the primary modelled QLF outcome because it combines fluorescence loss and LA and is a recognized QLF-derived measure of lesion burden within that domain.^22^^,^^23^ It was not treated as a biological gold standard or as a privileged reference measure, but rather as a structured, lesion-related endpoint for comparative modelling, representing one of several complementary outcomes that capture related but not identical aspects of lesion behaviour. Δ*Q* is not strongly aligned with all other QLF metrics across stages, and authors do not interpret this as a limitation of Δ*Q* itself, but as a reflection of the multidimensional nature of WSL assessment. Metrics such as Δ*F*, LA, and Δ*Q* capture different but related features of lesion change. The observed partial alignment among these measures therefore supports, rather than contradicts, the study’s central interpretation that no single parameter fully characterizes lesion dynamics.

The mixed-effects analyses further support this interpretation but also underscore the limited explanatory reach of any single measure. Only a subset of predictors showed significant associations with Δ*Q*, indicating that fluorescence-derived lesion-severity outcomes were only partially captured by the available optical and compositional indicators. Although Ca/P showed convergence with Δ*Q* at the correlation level, its association remained uncertain in mixed-effects models after accounting for within-tooth clustering. This does not argue against a biological relationship but rather suggests that localized near-surface compositional measurements alone may not linearly explain variation in fluorescence-derived lesion severity. Nonlinearity, modest effect sizes, and imperfect spatial correspondence between SEM–EDS sampling and lesion-wide optical outputs may all contribute to this pattern. Other QLF-derived metrics, including Δ*F* and LA, were also examined and explicitly reported at the correlation level. Accordingly, the modelling framework was not intended to establish a hierarchical or predictive relationship among methods, but to explore cross-method associations within a structured analytical context.

The model comparisons likewise support the multidomain nature of WSL assessment. The combined model outperformed single-domain models (clinical and compositional) in relative in-sample fit at both T1 and T3, as indicated by lower AIC/BIC values. However, this should not be interpreted as evidence of strong predictive performance. Rather, it indicates that integrating information across domains may improve interpretability within this dataset. The overall explanatory power remained modest, particularly at T3. In addition, several coefficient estimates were accompanied by wide confidence intervals, reflecting uncertainty in the model estimates. These results, therefore, support multimodal modelling primarily as an interpretive framework within this dataset rather than as a strong individual-level predictive tool. These features, together with the limited number of independent tooth-level units, suggest potential instability in model estimates and indicate that the model-based findings should be interpreted cautiously. The largely nonsignificant findings in group-stratified analyses further reinforce the fragility of single-variable associations once the data are partitioned by treatment condition. From a clinical perspective, the present findings do not support a one-size-fits-all measurement strategy for WSL monitoring. Rather, they suggest that different modalities may reflect different aspects of lesion behaviour, particularly across lesion formation and post-treatment stages. In the present dataset, intensity-based QLF parameters were more consistently associated with mineral-related measures after treatment, whereas LA appeared to provide complementary information on lesion extent. DIAGNOdent also showed directionally consistent associations with selected mineral-related indicators, but these findings should be interpreted cautiously and should not be taken as evidence of clinical superiority or as a basis for preferred monitoring strategies. Because this was an in vitro bovine model without a clinical reference standard, these observations are best viewed as hypothesis-generating and require clinical validation before informing routine practice.

These findings should be interpreted in light of several limitations. First, this was a secondary analysis of an in vitro bovine enamel dataset, and the number of independent experimental units was determined by the original tooth-level design rather than by a dedicated power calculation for the present intermethod analyses. Although the matched-block structure improved control of within-tooth variability and mixed-effects models accounted for clustering, the study may still have been underpowered to detect weaker cross-domain relationships, especially in stratified analyses where statistical power was further reduced. Moreover, because the treatment-stratified analyses were based on a very limited number of independent experimental units (*n* = 5 teeth per group), those findings should be interpreted with extreme caution. This limitation is also relevant for the mixed-effects modelling framework, as the small number of independent tooth-level units may have constrained the stability of parameter estimates and the ability to support more complex model structures. In addition, because random-slope structures could not be retained, the mixed-effects models were restricted to random intercepts. This may have limited the ability to capture between-tooth heterogeneity in intermethod slopes and should be considered when interpreting the possibility of model misspecification. Second, the bovine enamel model provided experimental standardization but does not fully reproduce the biological complexity of the oral environment, including saliva, pellicle formation, biofilm activity, and dynamic pH variation. Third, the compared modalities do not sample the same spatial domain. QLF and DIAGNOdent generate lesion-level optical signals influenced by subsurface structure and light propagation, whereas SEM–EDS provides localized near-surface elemental measurements. This spatial mismatch may itself contribute to weak cross-method association and limit direct one-to-one interpretation of optical vs compositional outputs. Finally, because the present framework distinguishes between intensity-related optical recovery and lesion-footprint reduction on an operational basis, these constructs should not be interpreted as directly validated biological compartments of remineralization. In addition, the modelling framework was not externally validated, and the reported model performance reflects in-sample associations only. Therefore, these findings should not be interpreted as evidence of predictive generalizability and require validation in independent datasets. Future studies should build on these findings by using larger datasets specifically designed for intermethod analyses, improving spatial correspondence across modalities, and validating in more clinically relevant settings.

## Conclusions

WSL assessment methods reflected complementary rather than interchangeable information. Association across modalities was limited after lesion induction, and stronger cross-method associations were observed after treatment, particularly between intensity-based QLF parameters and mineral-related measures. No single metric fully characterized WSL change, and the combined model provided only modest explanatory performance.

## Funding

This study was supported by the Ege University Department of University Research Projects (Project No: 23972; Tracking No: TS-DKT-2022-23972).

## Author contributions

ECF: the conception and design of the study, acquisition of data, interpretation of data, drafting the article, and final approval of the version to be submitted. GAS: analysis and interpretation of data, revising the article critically for important intellectual content, and final approval of the version to be submitted. BU: analysis and interpretation of data, revising the article critically for important intellectual content, and final approval of the version to be submitted. AME: the conception and design of the study, revising the article critically for important intellectual content, and final approval of the version to be submitted.

## Conflict of interest

The authors declare that they have no known competing financial interests or personal relationships that could have appeared to influence the work reported in this article.

## Appendix Supplementary materials

Image, application 1

## Footnotes

Supplementary material associated with this article can be found in the online version at doi:10.1016/j.identj.2026.109740.
