# Supplementary material for: Intermethod Association in Orthodontic White Spot Lesion Assessment: A Multimodal-Assessment
Source: Int Dent J. 2026 Jul 17;76(5):109740. doi: 10.1016/j.identj.2026.109740 (PMC13400400; doi:10.1016/j.identj.2026.109740)
Supplement: Supplementary file 1 [file mmc1.docx]

**Supplementary Table S1.** Stage-dependent changes in selected intermethod Spearman correlations between T1 and T3, assessed using Fisher's *r*-to-*z* transformation tests.

|  |  |  |  |  |  |
| --- | --- | --- | --- | --- | --- |
| Pair | **ρ (T1)** | **ρ (T3)** | **z** | **p (raw)** | **q (FDR)** |
| *Ca/P – ΔF* | −0.230 | 0.629 | −6.044 | < 0.001 | < 0.001 |
| *Ca/P – ΔQ* | 0.060 | 0.405 | −2.294 | 0.022 | 0.022 |
| *DIAGNOdent – ΔF* | 0.251 | −0.361 | 3.940 | < 0.001 | < 0.001 |
| *LA – Ca/P* | 0.024 | −0.433 | 3.024 | 0.003 | 0.003 |
|  |  |  |  |  |  |

FDR, false discovery rate (Benjamini–Hochberg procedure). *n* = 80 surfaces per time point. *z*, Fisher *z*-statistic for the difference between T1 and T3 Spearman correlation coefficients; negative *z* indicates the T3 correlation was larger in magnitude. All four pairs reached significance after FDR correction (*q* < 0.05).
